# miR-181a increases FoxO1 acetylation and promotes granulosa cell apoptosis via SIRT1 downregulation

**DOI:** 10.1038/cddis.2017.467

**Published:** 2017-10-05

**Authors:** Mei Zhang, Qun Zhang, Yali Hu, Lu Xu, Yue Jiang, Chunxue Zhang, Lijun Ding, Ruiwei Jiang, Jianxin Sun, Haixiang Sun, Guijun Yan

**Affiliations:** 1 Reproductive Medicine Center, The Affiliated Drum Tower Hospital of Nanjing University Medical School, Nanjing 210008, People’s Republic of China; 2 Center for Translational Medicine, Department of Medicine, Thomas Jefferson University, Philadelphia, PA, USA

## Abstract

Oxidative stress impairs follicular development by inducing granulosa cell (GC) apoptosis, which involves enhancement of the transcriptional activity of the pro-apoptotic factor Forkhead box O1 (FoxO1). However, the mechanism by which oxidative stress promotes FoxO1 activity is still unclear. Here, we found that miR-181a was upregulated in hydrogen peroxide (H_2_O_2_)-treated GCs and a 3-nitropropionic acid (NP)-induced *in vivo* model of ovarian oxidative stress. miR-181a overexpression promoted GC apoptosis, whereas knockdown of endogenous miR-181a blocked H_2_O_2_-induced cell apoptosis. Moreover, we identified that Sirtuin 1 (SIRT1), a deacetylase that suppresses FoxO1 acetylation in GCs, was downregulated by miR-181a and reversed the promoting effects of H_2_O_2_ and miR-181a on FoxO1 acetylation and GC apoptosis. Importantly, decreased miR-181a expression in the *in vivo* ovarian oxidative stress model inhibited apoptosis by upregulating SIRT1 expression and FoxO1 deacetylation. Together, our results suggest that miR-181a mediates oxidative stress-induced FoxO1 acetylation and GC apoptosis by targeting SIRT1 both *in vitro* and *in vivo*.

In mammals, the neonatal ovaries contain numerous resting primordial follicles, of which only a limited number successfully develop to ovulation, and more than 99% undergo degeneration at any stage following development. This process is termed follicular atresia and is regulated by many survival and pro-apoptotic factors.^1, 2, 3^ There is accumulating evidence that oxidative stress plays a vital role in the initiation of granulosa cell (GC) apoptosis in atretic follicles and is closely associated with ovarian aging and female subfertility.^4, 5, 6^

Forkhead box Os (FoxOs), a subfamily of forkhead box transcription factors, are known to play essential roles in many cellular processes, including proliferation, differentiation, and apoptosis. The FoxO family contains four members in mammals: FoxO1, FoxO3, FoxO4, and FoxO6. Three of these family members, FoxO1, FoxO3, and FoxO4, are expressed in the ovaries.^7, 8^ Among them, FoxO3, which is mainly expressed in oocytes of primordial follicles, negatively regulates oocyte growth.^9, 10^ In contrast, FoxO1 is highly expressed in GCs of atretic follicles.^11^ The overexpression of FoxO1 promotes GC apoptosis by activating the expression of pro-apoptotic factors FasL and Bim.^12, 13^ As a transcription factor, the activity of FoxO1 is primarily dependent on its phosphorylation and nuclear localization.^14, 15, 16^ Moreover, the transcriptional activity of FOXO proteins can also be regulated by acetylation and deacetylation. For example, p300 directly acetylates FoxO1 in the carboxyl-terminal region, stimulating FoxO1-induced transcription, and SIRT1/ SIRT2 has been shown to deacetylate FOXO1 and regulate its activity, especially under conditions of stress.^17, 18^ Recently, it was reported that oxidative stress induces FoxO1 nuclear translocation and activation in GCs, resulting in increased atretic follicles in mouse ovaries.^4^ However, the detailed mechanism remains unknown.

MicroRNAs (miRNAs) are highly conserved, small, non-coding RNAs that regulate gene expression at the post-transcriptional level through a complementary ‘seed sequence’.^19^ Dicer1, a ribonuclease III indispensable for mature miRNA synthesis, is expressed in oocytes and GCs. Conditional knockout of Dicer1 in GCs *in vivo* increases GC apoptosis and impairs mouse fertility, indicating that miRNAs may play vital roles in GC function.^20^ Recently, miRNAs in the ovary have been found to be involved in regulating GC proliferation (i.e., miR-224 and miR-145), differentiation (i.e., miR-224, miR-378, and miR-383), and apoptosis (i.e., miR-21, miR-23a, and miR-26b).^21^ Our previous study also demonstrated that miR-181a inhibits GC proliferation by binding to the 3′UTR of activin receptor 2A and repressing its expression.^22^ In addition, the level of miR-181a decreased in growing follicles compared with primary follicles in mice.^22^ These findings suggest that miR-181a may play important roles in ovarian follicular development.

In the present study, we found that miR-181a mediated H_2_O_2_-induced GC apoptosis *in vitro*. We also demonstrated that miR-181a increased FoxO1 acetylation by targeting SIRT1 expression both in apoptotic GCs and in a 3-nitropropionic acid (NP)-induced *in vivo* model of ovarian oxidative stress. Correspondingly, ectopic SIRT1 expression blocked H_2_O_2_- and miR-181a-induced FoxO1 acetylation and GC apoptosis. Our data highlight a functional role for miR-181a in ovarian follicular development and uncover a potential molecular target for the treatment of ovarian dysfunction.

## Results

### miR-181a mediates H_2_O_2_-induced GC apoptosis

**Figure 1:**
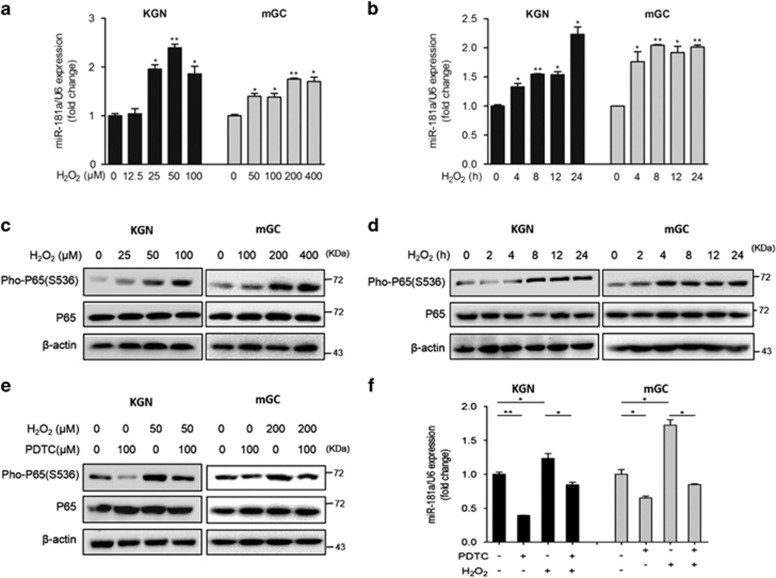
H_2_O_2_ promotes miR-181a expression through the activation of p65. (**a**) KGN cells and mGCs were treated with the indicated concentrations of H_2_O_2_ for 24 h. The levels of miR-181a were examined by qRT-PCR analysis. *P<0.05, **P<0.01 compared to 0 h. (**b**) KGN cells and mGCs were stimulated with 50 *μ*M H_2_O_2_ for 0, 4, 8, 12, or 24 h, as indicated. Changes in miR-181a expression were measured using a qRT-PCR assay. *P<0.05, **P<0.01 compared with the untreated control. (**c** and **d**) The dose- and time-dependent effects on p65 and pho-p65 expression in KGN cells and mGCs stimulated with H_2_O_2_ (50 *μ*M for KGN cells and 200 *μ*M for mGCs) were determined by western blot analysis. (**e**) GCs were pretreated with PDTC (100 *μ*M) or DMSO for 1 h before incubation with H_2_O_2_ (50 *μ*M for KGN cells and 200 *μ*M for mGCs) for 12 h. The levels of p65 and pho-p65 protein were determined by western blot analysis. (**f**) qRT-PCR analysis of miR-181a expression in KGN cells and mGCs after PDTC and/or H_2_O_2_ treatment. *P<0.05, **P<0.01 compared with the control group

H_2_O_2_ triggers oxidative stress-induced DNA damage, which leads to GC apoptosis through several pathways.^4^ To identify whether miRNA-mediated post-transcriptional gene regulation is involved in this process, KGN cells and mouse granulosa cells (mGCs) were treated with H_2_O_2_ at different concentrations for 24 h. miR-181a was upregulated ~2-fold over the endogenous level in KGN cells after 50 *μ*M H_2_O_2_ treatment and in mGCs exposed to 200 *μ*M H_2_O_2_ (Figure 1a), suggesting that KGN cells were more sensitive to H_2_O_2_ compared with mGCs. To further assess the effects of H_2_O_2_ on miR-181a expression patterns, we examined the miR-181a levels in KGN cells and mGCs stimulated with H_2_O_2_ (50 *μ*M for KGN cells and 200 *μ*M for mGCs) for 4–24 h using qRT-PCR. The results revealed that miR-181a was rapidly elevated at 4 h in both KGN cells and mGCs (Figure 1b). Previous studies have suggested that H_2_O_2_-induced oxidative stress initiates apoptosis and involves the NF-*κ*B signaling pathway,^23, 24^ and we observed that phosphorylated NF-*κ*B expression increased in a dose- and time-dependent manner in GCs following exposure to H_2_O_2_ (Figures 1c and d), consistent with a previous report.^25^ Moreover, when NF-*κ*B activation was inhibited by pretreatment with pyrrolidine dithiocarbamate (PDTC, an inhibitor of NF-*κ*B activation) (Figure 1e), the H_2_O_2_-induced increase in miR-181a expression was attenuated (Figure 1f), implying an essential role for activated NF-*κ*B in the upregulation of miR-181a expression under oxidative stress.

**Figure 2:**
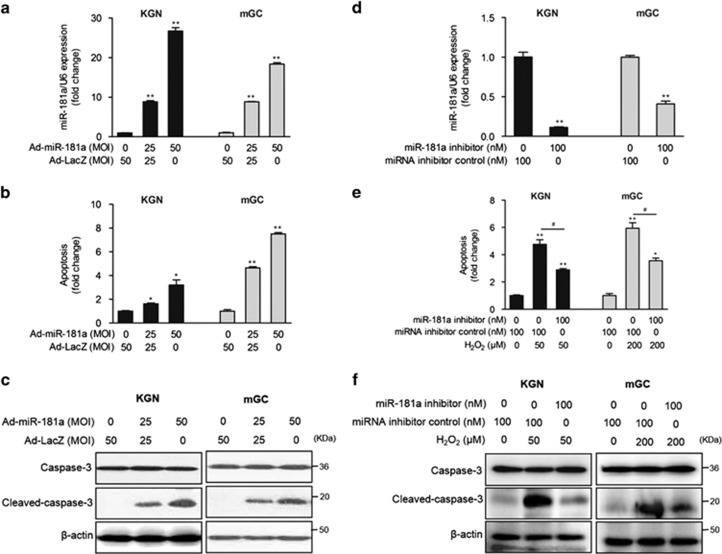
miR-181a promotes granulosa cell apoptosis. KGN cells and mGCs were infected with Ad-miR-181a (multiplicity of infection, MOI=0, 25, and 50) for 48 h. The levels of miR-181a (**a**) and internucleosomal DNA fragmentation (**b**) were detected by qRT-PCR and cell death detection assay, respectively. *P<0.05, **P<0.01 compared with the Ad-LacZ group. (**c**) Caspase-3 and cleaved caspase-3 levels were examined by western blot analysis. (**d**) KGN cells and mGCs were transfected with an miR-181a inhibitor or an miRNA inhibitor control (100 nM) for 36 h, and the cells were then treated with H_2_O_2_ (KGN with 50 *μ*M and mGCs with 200 *μ*M) for another 12 h. (**e**) Cell apoptosis was detected by a cell death detection assay. *P<0.05, **P<0.01 compared with the miRNA inhibitor control group. ^#^*P*<0.05 compared with the miR-181a inhibitor group. (**f**) Caspase-3 and cleaved caspase-3 levels were measured by western blot analysis

Furthermore, adenovirus-mediated miR-181a overexpression (Figure 2a) markedly promoted GC apoptosis and caspase-3 activation (a marker of apoptosis) in a dose-dependent manner based on cell death detection ELISA and western blotting analyses (Figures 2b and c). Notably, when endogenous miR-181a expression was knocked down in KGN cells and mGCs with a miR-181a inhibitor (Figure 2d) before H_2_O_2_ treatment, H_2_O_2_-enhanced DNA damage and activation of the apoptosis effector caspase-3 were attenuated (Figures 2e and f). Taken together, these observations demonstrate that miR-181a plays a positive role in H_2_O_2_-induced GC apoptosis.

### miR-181a promotes FoxO1 acetylation and nuclear localization in GCs

**Figure 3:**
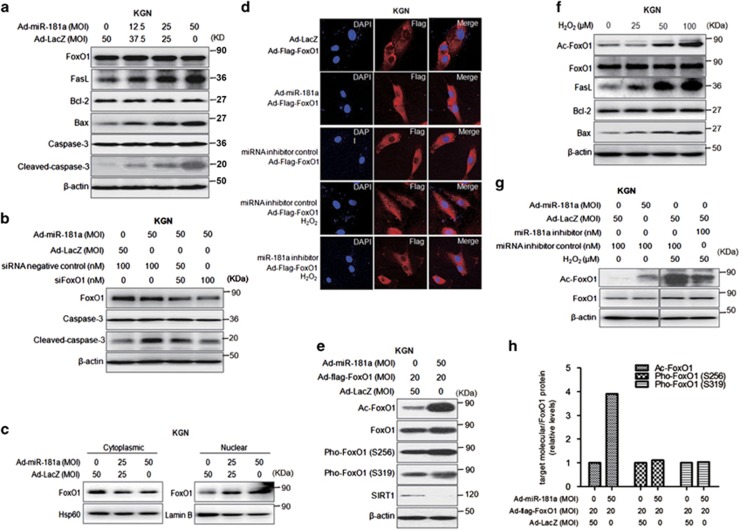
miR-181a promotes FoxO1 nuclear localization and FoxO1 acetylation in granulosa cells. (**a**) The total protein levels of FoxO1 and other proteins related to apoptosis in KGN cells were examined by western blotting after infection of Ad-miR-181a (MOI=0, 12.5, 25, and 50) for 48 h. (**b**) KGN cells were transfected with siRNA targeting FoxO1 for 12 h and then infected with Ad-miR-181a or Ad-LacZ, as indicated, for another 36 h. FoxO1, caspase-3 and cleaved caspase-3 levels were examined by western blot analysis. (**c**) KGN cells were infected with Ad-miR-181a (MOI=0, 25, and 50) for 48 h. The cytoplasmic and nuclear protein concentrations of endogenous FoxO1 were determined by western blot analysis. (**d**) An immunofluorescence assay was used to analyze the FoxO1 subcellular localization in KGN cells with the indicated treatment. FoxO1: red; DAPI: blue. (**e**) KGN cells were infected with Ad-flag-FoxO1 (MOI=20) alone or together with Ad-miR-181a as indicated for 48 h. The levels of FoxO1 acetylation and phosphorylation, total FoxO1, and SIRT1 were analyzed by western blot analysis. (**f**) KGN cells were treated with the indicated concentrations of H_2_O_2_ for 12 h. The protein levels of apoptosis-related genes and FoxO1 acetylation were measured by western blot analysis. (**g**) KGN cells were infected with Ad-miR-181a (MOI=50) for 48 h or transfected with an miR-181a inhibitor (100 nM) for 36 h followed by 50 *μ*M H_2_O_2_ treatment for another 12 h. Endogenous FoxO1 acetylation and total FoxO1 concentrations were examined by western blot analysis. (**h**) Quantification of the FoxO1 phosphorylation by making densitometric analysis on (**e**)

Since the transcription factor FoxO1 triggers GC apoptosis upon oxidative stress treatment, we examined endogenous FoxO1 expression in miR-181a-induced KGN cell apoptosis. As shown in Figure 3a, the total level of FoxO1 protein was not changed. However, FasL, a transcriptional target of FoxO1 that triggers apoptosis, was stimulated by miR-181a in a dose-dependent manner in addition to pro-apoptotic factor Bax expression and caspase-3 activation. Moreover, knockdown of endogenous FoxO1 protein in KGN cells (Figure 3b) and mGCs (Supplementary Figure S1A) substantially suppressed miR-181a-promoted caspase-3 cleavage (a marker of apoptosis).

We next examined the translocation of FoxO1 into the nucleus, a biochemical signature of H_2_O_2_-insulted GCs. We observed that ectopic miR-181a expression reduced the cytoplasmic level of FoxO1 and elevated the nuclear level of endogenous FoxO1 in a dose-dependent manner (Figure 3c). An immunofluorescence staining assay also showed that exogenous FoxO1 primarily localized to the cytoplasm of GCs and miR-181a overexpression induced FoxO1 nuclear translocation (Figure 3d and Supplementary Figure S1B). Moreover, loss of miR-181a efficiently blocked H_2_O_2_-induced FoxO1 nuclear accumulation (Figure 3d and Supplementary Figure S1B). Generally, FoxO1 subcellular localization is closely related to post-translational modifications, including phosphorylation and acetylation. To determine the mechanism of FoxO1 subcellular translocation, we examined the phosphorylation level of FoxO1 in KGN cells infected with Ad-flag-FoxO1 alone or together with Ad-miR-181a for 48 h. There was no obvious change in FoxO1 phosphorylation at either-Ser256 or Ser319 sites that can be phosphorylated by PI3K/Akt and promote FoxO1 nuclear export. Conversely, miR-181a overexpression remarkably increased FoxO1 acetylation about four-fold (Figures 3e and h). Interestingly, SIRT1, a deacetylase responsible for FoxO1 deacetylation,^26^ was downregulated by miR-181a (Figure 3e). Importantly, we also found that H_2_O_2_ enhanced endogenous FoxO1 acetylation in a dose-dependent manner, which coincided with increased FasL and Bax expression in KGN cells. However, the total FoxO1 level and Bcl-2 expression were not altered by H_2_O_2_ treatment (Figure 3f). In addition, knockdown of miR-181a weakened H_2_O_2_-induced FoxO1 acetylation in both KGN cells and mGCs (Figure 3g and Supplementary Figure S1C). These findings strongly suggest that FoxO1 acetylation is involved in H_2_O_2_- and miR-181a-induced GC apoptosis.

### miR-181a promotes GC apoptosis via modulation of SIRT1-dependent FoxO1 deacetylation in GCs

**Figure 4:**
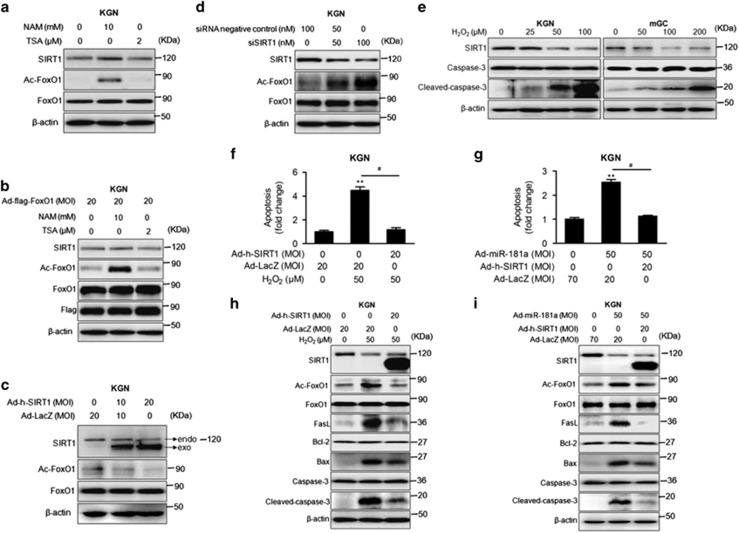
SIRT1 deacetylates FoxO1 in granulosa cells. (**a** and **b**) KGN cells were infected with or without Ad-flag-FoxO1 (MOI=20) for 12 h, followed by 10 mM NAM or 2 *μ*M TSA treatment for another 12 h. The levels of SIRT1, acetylated FoxO1, and total FoxO1 were measured. (**c**) KGN cells were infected with Ad-h-SIRT1 (MOI=0, 10, and 20) for 48 h. Western blotting was performed to measure the protein levels of SIRT1, acetylated FoxO1, and total FoxO1. (**d**) Western blotting was performed to analyze SIRT1, acetylated FoxO1, and total FoxO1 in KGN cells transfected with siRNA targeting SIRT1 (0, 50, or 100 nM) for 48 h. (**e**) KGN cells or mGCs were treated with H_2_O_2_ at different concentrations, as indicated. The expression patterns of SIRT1, caspase-3, and cleaved caspase-3 were examined by western blot analysis. KGN cells were infected with Ad-h-SIRT1 (MOI=20) for 36 h, followed by H_2_O_2_ treatment (50 *μ*M) for another 12 h. (**f**) Cell apoptosis was analyzed by a cell death detection assay. **P<0.01, ^#^*P*<0.05 compared with the control group. (**h**) The protein levels of SIRT1, acetylated FoxO1, total FoxO1, and apoptosis-related genes were determined by western blot analysis. KGN cells were infected with Ad-miR-181a and/or Ad-h-SIRT1, as indicated, for 48 h. (**g**) Cell apoptosis was analyzed by a cell death detection assay. **P<0.01, ^#^*P*<0.05 compared with the control group. (**i**) The protein levels of SIRT1, acetylated FoxO1, total FoxO1, and apoptosis-related genes were determined by western blot analysis

To elucidate the functional target that mediates the effect of miR-181a on FoxO1 acetylation and activation, we first demonstrated that miR-181a directly bound to and downregulated SIRT1 expression in KGN cells and mGCs (Supplementary Figure S2). Subsequently, we found that the acetylation of endogenous and exogenous FoxO1 was induced by nicotinamide (NAM), which blocks SIRT1 activity in KGN cells.^27^ Contrarily, trichostatin A (TSA), an inhibitor of class I and II histone deacetylases (HDACs), had no obvious effect on FoxO1 acetylation (Figures 4a and b). Moreover, SIRT1 overexpression in KGN cells decreased the level of acetylated FoxO1 (Figure 4c), and siRNA-mediated knockdown of endogenous SIRT1 protein expression significantly promoted FoxO1 acetylation in KGN cells (Figure 4d) and mGCs (Supplementary Figures S3A and B). We also found that the level of acetylated FoxO1 in primary mGCs was decreased by SIRT1 activator 3 (SA3) treatment (Supplementary Figure S3C). Collectively, these data suggest that SIRT1 can mediate FoxO1 deacetylation in GCs.

In addition, SIRT1 expression was dose-dependently reduced by H_2_O_2_ in KGN cells and mGCS, suggesting that SIRT1 may be a functional target of miR-181a in the regulation of H_2_O_2_-induced GC apoptosis (Figure 4e). As we expected, ectopic SIRT1 expression in KGN cells reversed the promoting effects of H_2_O_2_ on DNA damage in KGN cells (Figure 4f). Consistently, H_2_O_2_-induced FoxO1 acetylation and pro-apoptotic gene activation were also blocked by SIRT1 overexpression in KGN cells (Figure 4h). Moreover, miR-181a-induced cell apoptosis and pro-apoptotic signaling pathway activation were similarly weakened by SIRT1 (Figures 4g and i). In primary mGCs, SA3 also inhibited miR-181a-induced cell apoptosis (Supplementary Figure S3D), FoxO1 acetylation and caspase-3 activation (Supplementary Figure S3E). Consequently, we concluded that the downregulation of SIRT1 by miR-181a mediates H_2_O_2_-induced FoxO1 acetylation and GC apoptosis.

### miR-181a-induced FoxO1 acetylation and GC apoptosis in the ovary

**Figure 5:**
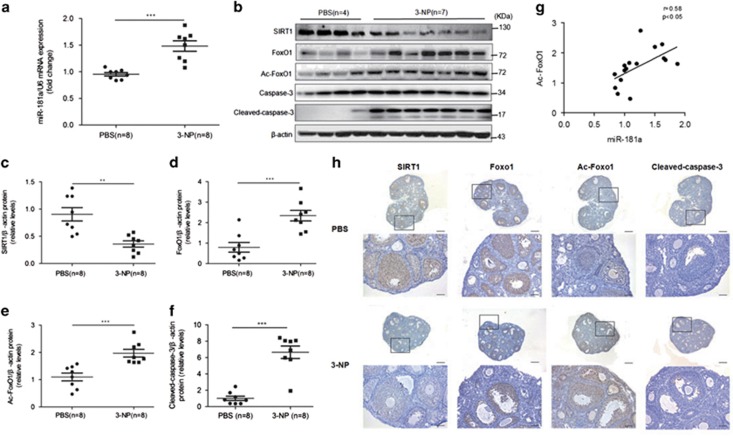
Activation of the miR-181a-SIRT1-FoxO1 apoptotic pathway in the *in vivo* ovarian oxidative stress model. Female 4-week-old ICR mice were injected with 50 mg/kg 3-NP (*n*=8) or PBS (*n*=8) twice daily for 5 days. (**a**) The miR-181a levels in ovaries were determined by qRT-PCR analysis. ***P<0.001 compared with the PBS control group. (**b**) Total protein lysates from ovarian tissue samples (*n*=11) were subjected to western blot analysis and immunoprobed with antibodies specific to SIRT1, FoxO1, acetylated FoxO1, caspase-3, and cleaved caspase-3. *β*-actin was used as a loading control. Protein expression levels were normalized to *β*-actin. The data for all the samples from the PBS control group (*n*=8) and the 3-NP group (*n*=8) are shown in the scatter plots (**c**–**f**). *P<0.05, **P<0.01 compared with the PBS control group. (**g**) Correlations between miR-181a and acetylated FoxO1 expression in GCs from the PBS and 3-NP groups. (**h**) Immunostaining for SIRT1, FoxO1, acetylated FoxO1, and cleaved caspase-3 in ovaries from the PBS and 3-NP groups. Nonspecific rabbit IgG was used as a negative control. Brown represents positive staining. Scale bar, 50 *μ*m

To validate the role of miR-181a-induced FoxO1 acetylation and GC apoptosis *in vivo*, oxidative stress-induced GC apoptosis was examined in 3-NP-injected mice. As shown in Figure 5a, qRT-PCR revealed a significant increase in miR-181a expression in the 3-NP-treated ovary compared with the phosphate-buffered saline (PBS) control. The administration of 3-NP also produced a significant stimulatory effect on FoxO1, Ac-FoxO1, and SIRT1 expression in the ovary and GCs relative to the control, which was consistent with the *in vitro* results from western blotting (Figures 5b–f) and immunohistochemical analyses (Figure 5h). Furthermore, we observed a significant positive correlation between miR-181a and Ac-FoxO1 expression (Figure 5g), confirming the promotive effect of miR-181a on the oxidative stress-induced apoptosis of GCs.

**Figure 6:**
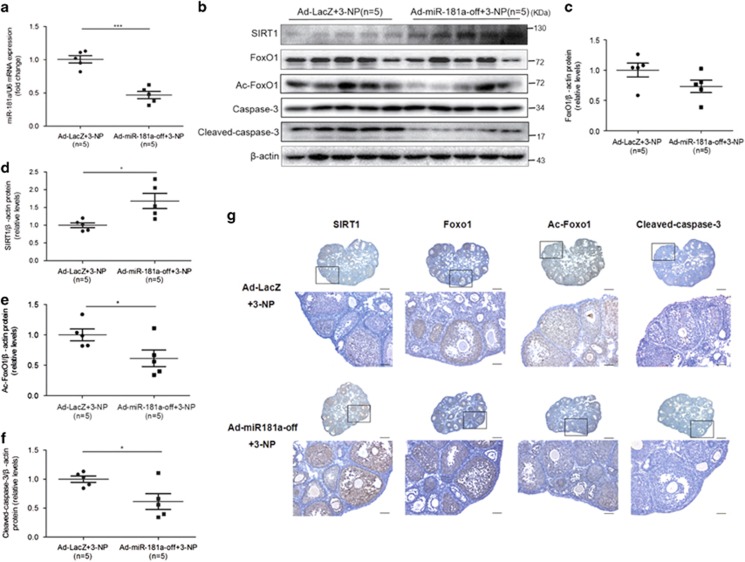
Knockdown of endogenous miR-181a blocked apoptotic pathway activation in the oxidative stress model. Paired ovaries of 28-day-old ICR female mice were excised, cultured *in vitro*, and infected with Ad-LacZ and AdmiRa-mmu-miR-181a-off adenovirus (2.5 × 10^10^pfu/ovary) for another 48 h before being stimulated with 3-NP. (**a**) qRT-PCR analysis of miR-181a expression in ovaries (*n*=5 ovaries per group). ***P<0.001 compared with the Ad-LacZ+3-NP group. (**b**–**f**) The protein levels of SIRT1, FoxO1, acetylated FoxO1, caspase-3, and cleaved caspase-3 in ovarian tissue were determined by western blot analysis and were normalized to *β*-actin. *P<0.05 compared with the Ad-LacZ+3-NP group. (**g**) Immunostaining for SIRT1, FoxO1, acetylated FoxO1, and cleaved caspase-3 in cultured ovaries *in vitro*. Nonspecific rabbit IgG was used as a negative control. Brown represents positive staining. Scale bar, 50 *μ*m

Next, we knocked down miR-181a expression by infecting cultured 28-day-old mouse ovaries miR-181a-off adenovirus and a LacZ control adenovirus *in vitro* to investigate the effect of reduced miR-181a expression on 3-NP-induced GC apoptosis in the ovary (Figure 6a). We found that miR-181a knockdown was associated with the subsequent upregulation of SIRT1 and significant reductions in FoxO1 acetylation and cleaved caspase-3 expression relative to the control (Figures 6b–g). Together, these data demonstrated that miR-181a induces GC apoptosis by inhibiting SIRT1-mediated deacetylation of FoxO1 *in vivo.*

## Discussion

Oxidative stress, one of the most important inducers of cellular damage involved in female reproduction,^26^ increases in atretic follicles and initiates GC apoptosis.^4, 5, 6, 28^ Inhibition of oxidative stress protects GCs from apoptosis both *in vitro* and *in vivo*.^29, 30^ Despite the necessity of FoxO1 transcriptional activation for oxidative stress-induced GC apoptosis,^4^ little is known about the detailed mechanism. Herein, we identified a single miRNA, miR-181a, which can modulate the SIRT1-mediated deacetylation of FoxO1 and promote GC apoptosis both *in vitro* and in a 3-NP-induced *in vivo* model of ovarian oxidative stress.

Several miRNAs have been reported to function in GC apoptosis and follicular atresia. For example, miR-21, an LH-induced miRNA, blocks GC apoptosis.^31^ miR-23a and miR-26b, which are highly expressed in atretic follicles, promote GC apoptosis by inhibiting XIAP and ATM, respectively.^32, 33^ Recently, greater focus has been placed on the effects of miRNAs, such as miR-204 and miR-133, on H_2_O_2_-induced apoptosis.^34, 35^ In the present study, we found that the level of miR-181a was significantly increased in GCs upon treatment with H_2_O_2_, while gain- and loss-of-function experiments showed that miR-181a mediated oxidative stress-induced GC apoptosis both *in vitro* and *in vivo*. Increasing literatures believe that NF-*κ*B can modulate several miRNAs expression to participate in cell proliferation and growth;^36, 37^ our results show that the increase in miR-181a expression induced by H_2_O_2_ stimulation was attenuated when pretreated with PDTC, implying that miR-181a is a new member in the pathway between NF-*κ*B and H_2_O_2_-induced apoptosis. Thus, our study not only identifies a new miRNA that regulates GC apoptosis but also provides detailed insight into the mechanisms involved in the process of oxidative stress-induced GC apoptosis. Considering that GC proliferation is also suppressed by miR-181a,^22^ we propose that increased miR-181a expression in ovarian GCs may be a cause of ovarian dysfunction via inhibited follicular growth or accelerated follicular degeneration. In the future, we will further investigate the role of miR-181a in follicular development *in vivo* using GC-specific miR-181a knock-in mice.

In ovaries, the transcription factor FoxO1 is primarily expressed in apoptotic GCs.^11^ FoxO1 triggers GC apoptosis by enhancing FasL and Bim expression upon oxidative stress treatment, which activates the mitochondrial apoptosis pathway by elevating the Bax/Bcl-2 ratio, leading to caspase-3 cleavage.^4, 12, 13, 38^ FoxO1 has been revealed to be negatively regulated by several miRNAs through directly binding to its 3′UTR.^39, 40^ In the present study, we also found that miR-181a and H_2_O_2_ induced GC apoptosis by inducing FoxO1 nuclear accumulation instead of altering the FoxO1 protein level. It is well known that PI3K/Akt-mediated FoxO1 phosphorylation is a cause of FoxO1 nuclear export.^14, 15, 16^ However, we also observed no obvious change in FoxO1 phosphorylation in GCs with Ad-miR-181a or H_2_O_2_ administration, consistent with previous study.^41^ Instead, FoxO1 acetylation was dramatically induced by miR-181a and H_2_O_2_. All of these findings lead us to speculate that miR-181a mediates H_2_O_2_-induced GC apoptosis by increasing the FoxO1 acetylation/nuclear import pathway instead of decreasing the FoxO1 phosphorylation/nuclear exclusion pathway.

FoxO acetylation is well known to be regulated by HDACs. For example, class I HDACs regulate FoxO acetylation in muscle atrophy.^42^ Class II HDACs function in mammalian glucose homeostasis through controlling FoxO acetylation.^43^ However, bioinformatics-based analyses have revealed that no predicted seed sequences of miR-181a are present in the 3′ UTRs of any class I or II HDAC genes. SIRT1, an NAD^+^-dependent class III deacetylase, promotes gene transcription by deacetylating specific transcription factors, including FoxO1.^18, 44, 45^ The interaction between FoxO1 and SIRT1 can be enhanced by FHL2 and impaired by FCoR, resulting in decreased and increased FoxO1 transcriptional activity, respectively.^18^ SIRT1 has been reported to be reduced upon H_2_O_2_ treatment, and the SIRT1 activator resveratrol mediated the protective effects of vitamin C against H_2_O_2_ in a human retinal pigmented epithelial cell line.^46^ Recently, SIRT1 was revealed to have potential roles in ovarian function.^47, 48, 49^ For example, the overexpression of SIRT1 increased proliferation marker gene expression and progesterone release.^47^ The SIRT1 activator resveratrol played a protective role in age-associated infertility in mice, and SIRT1 signaling protected oocytes from oxidative stress-induced damage.^48, 49^ However, the mechanisms modulating SIRT1 expression and the function of SIRT1 in GC apoptosis have not been confirmed. In this study, we found that SIRT1 deacetylated FoxO1 in GCs, which was suppressed by both miR-181a and H_2_O_2_ treatment. A targeting study demonstrated that miR-181a suppressed SIRT1 expression at both the transcriptional and translational levels by binding to the 3′ UTR of SIRT1, which is consistent with a previous study.^50^ SIRT1 activation blocked miR-181a- and H_2_O_2_-induced pro-apoptotic pathway activation and GC apoptosis *in vitro*. In addition, we further demonstrated the functional role of miR-181a-induced FoxO1 acetylation and GC apoptosis using a 3-NP-induced *in vivo* model of ovarian oxidative stress, suggesting the miR-181a/SIRT1/FoxO1 pathway as a potential target for preventing and treating follicular atresia.

In conclusion, our findings imply that the upregulation of miR-181a mediates FoxO1 nuclear accumulation in oxidative stress-induced GC apoptosis as a result of decreased SIRT1 expression and increased FoxO1 acetylation. Revealing the critical role of miR-181a in oxidative stress-induced FoxO1 activation and GC apoptosis more clearly elucidates the mechanism of follicular atresia, which is a key pathological process in ovarian dysfunction and infertility.

## Materials and methods

### Mice

Three- or four-week-old Institute of Cancer Research (ICR) mice were purchased from the Experimental Animal Center of Yangzhou University (Yangzhou, China) and maintained in the Animal Laboratory Center of Nanjing Drum Tower Hospital (Nanjing, China) on a 12/12 h light/dark cycle (lights off at 1900 hours) with food and water available *ad libitum*. All experiments involving animals were approved by the Institutional Animal Care and Use Committee of Nanjing Drum Tower Hospital (SYXK 2014-0052).

### Granulosa cell isolation and culture

For primary mGC isolation, ovaries were removed from 21-day-old immature ICR mice and were punctured with 25-gauge needles. The cells were pooled and filtered with a 40-*μ*m cell strainer to remove oocytes. The isolated cells were determined to have 95% purity via follicle-stimulating hormone receptor staining as described previously.^22^ The mGCs were cultured in DMEM/F12 medium (Gibco BRL/Invitrogen, Carlsbad, CA, USA) containing 10% fetal bovine serum (HyClone, South Logan, UT, USA), 1 mM sodium pyruvate, 2 mM glutamine, 100 IU/ml penicillin, and 100 *μ*g/ml streptomycin. KGN cells, a human ovarian GC-like tumor cell line, were maintained in DMEM/F12 medium (Gibco BRL/Invitrogen) supplemented with 10% newborn calf serum (Gibco BRL/Invitrogen), 100 IU/ml penicillin, and 100 *μ*g/ml streptomycin. All cells were maintained at 37 °C in a humidified environment with 5% CO_2_. In some experiments, GCs were cultured in the presence of H_2_O_2_ (Sigma, St. Louis, MO, USA), nicotinamide (NAM; Sigma), trichostatin A (TSA; Sigma), or SIRT1 activator 3 (SA3; Santa Cruz Biotechnology, Santa Cruz, CA, USA).

### Establishment of ovarian oxidative stress mouse model

The ovarian oxidative stress mouse model was established as previously described.^51^ Briefly, female 4-week-old ICR mice were used in the experiments, and 3-NP (Sigma-Aldrich, St. Louis, MO, USA) was dissolved in PBS to a concentration of 10 mg/ml (pH 7.4) and administered by intraperitoneal injection at a dose of 50 mg/kg twice daily for 5 days at 12 h intervals (800 and 2000 hours). At 12 h after the last injection, the mice were killed, and paired ovaries were excised and washed three times with PBS. The left ovary of each mouse was flash-frozen and stored at −80 °C to assess the mRNA and protein levels, and the right ovary of each mouse was fixed in buffered formalin for immunohistochemical analysis.

### *In vitro* culture of mouse ovaries

Paired ovaries of 28-day-old ICR female mice were excised and washed three times in Waymouth’s medium containing 3 mg/ml BSA. Ovaries (paired ovaries/well) were cultured on Millicell inserts (Millipore, Billerica, MA, USA) in 24-well plates (Corning, NY, USA) with 600 *μ*l of Waymouth’s MB752/1 medium (Gibco) supplemented with 1 mM sodium pyruvate, 100 IU/ml penicillin, 100 *μ*g/ml streptomycin, 3 mg/ml BSA, 10% FBS, and 0.6 IU/ml rFSH (Merck, Germany). After 24 h of culture, the ovaries were infected with Ad-LacZ and AdmiRa-mmu-miR-181a-off adenovirus (2.5 × 10^10^ pfu/ovary) for another 48 h before the medium was changed. The ovaries were then cultured with 2 *μ*g/ml 3-NP for an additional 48 h at 37 °C in a humidified atmosphere of 5% CO_2_. The cultured ovaries were collected for measurements of mRNA and protein expression.

### Generation of recombinant adenovirus

An adenovirus vector harboring the full-length FoxO1 with a Flag tag before the sequence (Ad-flag-FoxO1) and Ad-miR-181a were generated using the AdMax (Microbix Biosystems, Inc., Toronto, Canada) and pSilencer adeno 1.0-CMV systems (Ambion, Austin, TX, USA) according to the manufacturer’s instructions as previously described.^22^ The human SIRT1 adenovirus (Ad-h-SIRT1) and AdmiRa-mmu-miR-181a-off adenovirus were purchased from Applied Biological Materials Inc. (Richmond, Canada). The adenovirus-bearing LacZ (Ad-LacZ) was obtained from Clontech (Palo Alto, CA, USA) and was used as a control in the adenovirus-mediated miR-181a, FoxO1, and SIRT1 overexpression experiments. The virus was packaged and amplified in HEK293A cells and purified using CsCl banding.

### Oligonucleotide transfection

The miR-181a inhibitor (2′-Omethyl-modified antisense oligonucleotides specifically targeting mature miR-181a), miRNA inhibitor negative control, siRNA targeting FoxO1 (human, 5′-GGACAACAACAGUAAAUUUdTdT-3′ mouse, 5′-CCGCCAAACACCAGUCUAAdTdT-3′), siRNA targeting SIRT1 (human, 5′-GCUAAGAAUUUCAGGAUUAdTdT-3′ mouse, 5′-CCAUGAAGUGCCUCAAAUAdTdT-3′), and siRNA negative control were all synthesized by Ribobio (Guangzhou, China). For loss-of-function experiments, these oligonucleotides were transfected into GCs using Lipofectamine 2000 (Invitrogen, Carlsbad, CA, USA). Neither the miRNA inhibitor negative control nor the siRNA negative control shared homologous regions with the human or mouse genome sequences.

### RNA isolation and quantitative real-time PCR

Total RNA was extracted from cells or ovarian tissue using Trizol reagent (Life Technologies). Subsequently, 2 *μ*g of RNA was reverse-transcribed into cDNA using the PrimeScript RT reagent kit (Bio-Rad, Hercules, CA, USA), and quantitative real-time PCR (qRT-PCR) was performed on a MyiQ Single-Color Real-Time PCR Detection System (Bio-Rad). The miR-181a and mRNA expression levels were normalized to U6 small nuclear RNA and 18S, respectively, using the 2^−△△CT^ method as previously described.^22^ The specific primer sequences are listed in Table 1.

### Cell death detection assay

Cells were seeded into 12-well plates (10^5^ cells/well), and cell apoptosis was assessed using the Cell Death Detection ELISA kit (Roche Molecular Biochemicals, Mannheim, Germany) according to the manufacturer’s instructions. Briefly, cells were first lysed using the lysis buffer at room temperature for 30 min before being centrifuged for 10 min. Then, 20 *μ*l of the supernatant was used to examine the level of internucleosomal DNA fragmentation. Finally, the absorbance at 405 nm and 490 nm was measured using a 96-well plate reader (Thermo Electron Corp, Taunton, MA, USA). The relative cell death was determined according to the absorbance values.

### Western blotting and extraction of nuclear and cytoplasmic protein

Total protein was prepared from cultured GCs or ovarian tissue as previously described.^19^ Nuclear and cytoplasmic proteins were extracted using the NE-PER nuclear and cytoplasmic extraction reagents (Thermo) according to the manufacturer’s instructions. Immunoblotting was performed with primary antibodies against caspase-3 (1 : 1000; Cell Signaling Technology, #9662s, Danvers, MA, USA), cleaved caspase-3 (1 : 500; Cell Signaling Technology, #9661s), FoxO1 (1 : 1000; Cell Signaling Technology, #2880), Ac-FKHR (1 : 500; Ac-FoxO1, Santa Cruz Biotechnology, sc-49437), phospho-FoxO1 (Ser 256, 1 : 1000; Cell Signaling Technology), phospho-FoxO1 (Ser 319, 1 : 1000; Bioworld Technology, BS4712, MN, USA), FasL (1 : 500; Bioworld Technology, BS1122), Bcl-2 (1 : 1000; Proteintech, 12789-1-AP, Wuhan, China), Bax (1 : 500; Bioworld Technology, BS6420), and SIRT1 (1 : 500; Santa Cruz Biotechnology, sc-15404). *β*-actin (1 : 10000; Abcam, Cambridge, CA, USA), HSP60 (1 : 1000; Bioworld Technology), and Lamin B (1 : 1000; Santa Cruz Biotechnology, sc-6216) served as internal controls for detecting the expression levels of total protein lysates, cytoplasmic protein lysates, and nuclear protein lysates, respectively.

### Luciferase reporter assay

Based on the human and mouse SIRT1 mRNA sequences in GenBank (accession nos. NM_012238.4 and NM_019812.2), the 3′-UTR of the human SIRT1 gene (nt 1 to 397) or mouse SIRT1 gene (nt 1 to 339) was amplified separately from the genomic DNA of human and mouse GCs and was cloned into a pGL3-promoter luciferase reporter vector using *Xba*I restriction sites. The primers for the 3′-UTRs of SIRT1 were (human) 5'-CGCGTCTAGATGTAACAATTGTGCAGGTAC-3' and 5'-CGCGTCTAGATTAGCTGTTCCCTTTACATT-3' and (mouse) 5'-CGTATCTAGACACTATTGAAGCTGTCCGGA-3' and 5'-CCGGTCTAGATAAGCTGTTCCCTTTACATT-3'. Pre-confluent (60–70%) KGN cells or mGCs in 12-well plates were infected with Ad-miR-181a and transfected with 300 ng of a firefly luciferase reporter plasmid and 20 ng of the pRL-RSV Renilla luciferase reporter plasmid using Lipofectamine 2000. After 48 h, the cells were lysed, and the luciferase activity assay was performed as previously described.^22^

### Immunofluorescence staining

GCs were cultured in eight-well chambers (Millipore, Billerica, MA, USA) and either infected with Ad-miR-181a/Ad-flag-FoxO1 for 48 h or treated with miR-181a inhibitor/Ad-flag-FoxO1 for 36 h, followed by H_2_O_2_ treatment for another 12 h. The cells were then washed with PBS, fixed with 4% paraformaldehyde for 30 min at room temperature, permeabilized with 0.5% Triton X-100 in PBS, and incubated with anti-FoxO1 antibody (Cell Signaling Technology) at 4 °C overnight. The following day, the cells were incubated with Alexa Fluor 594-conjugated donkey-anti-rabbit IgG (Life Technology) for 1 h at 37 °C in the dark. The cell nuclei were stained with DAPI (5 *μ*g/ml). Finally, the images were visualized using a laser-scanning confocal microscope (Leica, Germany).

### Immunohistochemicals staining

Ovarian tissue sections were immunostained with primary antibodies against FoxO1 (1 : 250, Cell Signaling Technology), SIRT1 (1 : 250; Santa Cruz Biotechnology), Ac-FoxO1 (1 : 400; Santa Cruz Biotechnology), and cleaved caspase-3 (1 : 200; Cell Signaling Technology) overnight at 4 °C. The following day, the sections were incubated with a goat anti-rabbit secondary antibody (rabbit ABC detect kit, ZSBio, Beijing, China) at 37 °C for 30 min. Next, the sections were stained with 3, 3'-diaminobenzidine (DAB) and counterstained with hematoxylin. Control sections were similarly pretreated and processed concurrently with the experimental sections using nonspecific rabbit IgG. Nonspecific staining was not detected in the controls.

### Statistical analysis

In this study, each experiment was performed at least three times. The data are presented as the mean±standard deviation (S.D.). The statistical analysis consisted of an ANOVA followed by the Student–Newman–Keuls test for experiments involving more than two groups. Student’s *t-*test was performed for comparisons of two groups. *P-*values <0.05 were considered statistically significant.

## Figures and Tables

**Table 1:** Oligonucleotide primer sequences of quantitative real-time PCR

**Name**	**Forward primer (5′→3′)**	**Reverse primer (5′→3′)**
miR-181a	ACACTCCAGCTGGGAACATTCAACGCTGTCG	GGTGTCGTGGAGTCGGCAATTCAGTTGAG
U6	CTCGCTTCGGCAGCACA	AACGCTTCACGAATTTGCGT
hSIRT1	CAGGTTGCGGGAATCCAAAG	GCTGGGCACCTAGGACATCG
h18S	CGGCTACCACATCCAAGGAA	CTGGAATTACCGCGGCT
mSIRT1	AGTTCCAGCCGTCTCTGTGT	CTCCACGAACAGCTTCACAA
m18S	ATGGCCGTTCTTAGTTGGTG	CGGACATCTAAGGGCATCAC
