# Supplementary material for: miR-181a increases FoxO1 acetylation and promotes granulosa cell apoptosis via SIRT1 downregulation
Source: Cell Death Dis. 2017 Oct 5;8(10):e3088–. doi: 10.1038/cddis.2017.467 (PMC5680589; doi:10.1038/cddis.2017.467)
Supplement: Supplementary Information [file cddis2017467x1.docx]

**Figure S1.** **miR-181a promotes FoxO1 nuclear localization and FoxO1 acetylation in mGCs.** (A) mGCs were transfected with siRNA targeting FoxO1 for 12 h and then infected with Ad-miR-181a or Ad-LacZ, as indicated, for another 36 h. FoxO1, caspase-3, and cleaved caspase-3 levels were examined by western blot analysis. (B) An immunofluorescence assay was used to analyze the FoxO1 subcellular localization in mGCs with the indicated treatment. FoxO1: red; DAPI: blue. (C) mGCs were infected with Ad-miR-181a (MOI=50) for 48 h or were transfected with an miR-181a inhibitor (100 nM) for 36 h, followed by 200 µM H_2_O_2_ treatment for another 12 h. Endogenous FoxO1 acetylation and total FoxO1 concentrations were examined by western blot analysis.

**Figure S2.** **miR-181a negatively modulates SIRT1.** (A) Putative binding sites for human (hsa) miR-181a and mouse (mmu) miR-181a in the 3’ -UTR of the SIRT1 gene. (B) KGN cells and mGCs were infected with Ad-miR-181a and transfected with the luciferase-SIRT1-3’ UTR constructs, as indicated. After 48 h, luciferase assays were performed. (C and D) KGN cells and mGCs were infected with Ad-miR-181a (MOI=50) or were transfected with miR-181a inhibitor (100 nM), as indicated, for 48 h. The protein expression and mRNA expression of SIRT1 were evaluated by western blotting and qRT-PCR analysis, respectively. *p<0.05, **p<0.01 compared to the control group.

**Figure S3.** **miR-18 1a/SIRT1 pathway controls the acetylation of FoxO1 in mGCs.** (A and B) mGCs were transfected with SIRT1 siRNA (0, 100, or 200 nM) for 24 h, followed with or without Ad-flag-FoxO1 (MOI=20) infection for another 24 h. The levels of SIRT1, acetylated FoxO1, and total FoxO1 were examined by western blotting. (C) mGCs were treated with SA3 as indicated for 24 h, and western blotting was used to examine the expression levels of SIRT1, acetylated FoxO1, and total FoxO1. mGCs were treated with 50 MOI Ad-miR-181a and/or 5 µM SA3 for 48 h. (D) Cell apoptosis was analyzed by a cell death detection assay. **p<0.01, ^##^p<0.01 compared with the control group. (E) Total and acetylated FoxO1, caspase-3, and cleaved caspase-3 levels were evaluated by western blot assay.
