# Supplementary figures and images for: miR-181a increases FoxO1 acetylation and promotes granulosa cell apoptosis via SIRT1 downregulation
Source: Cell Death Dis. 2017 Oct 5;8(10):e3088–. doi: 10.1038/cddis.2017.467 (PMC5680589; doi:10.1038/cddis.2017.467)

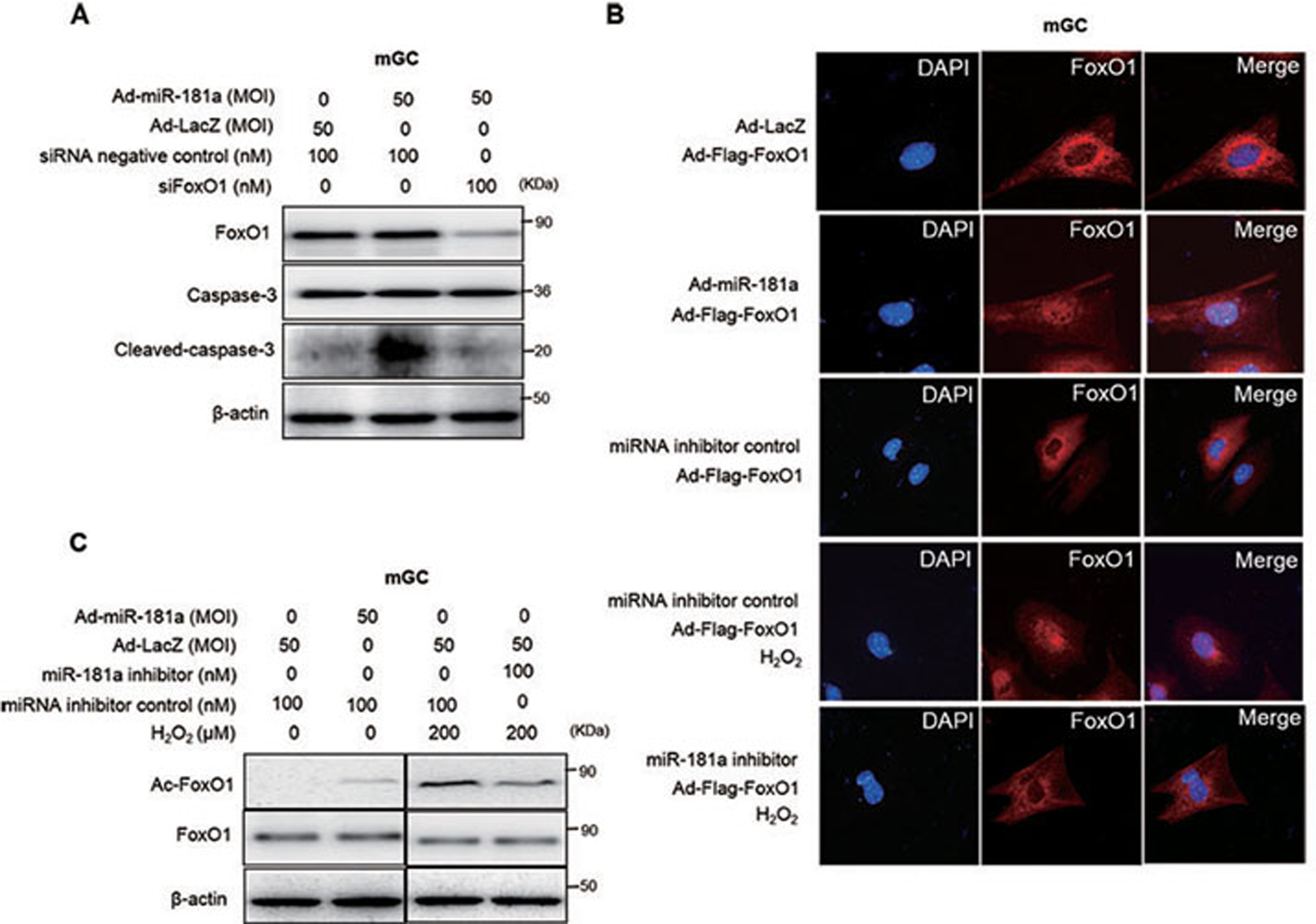

Supplement: Supplementary Figure S1 [file cddis2017467x2.tif]

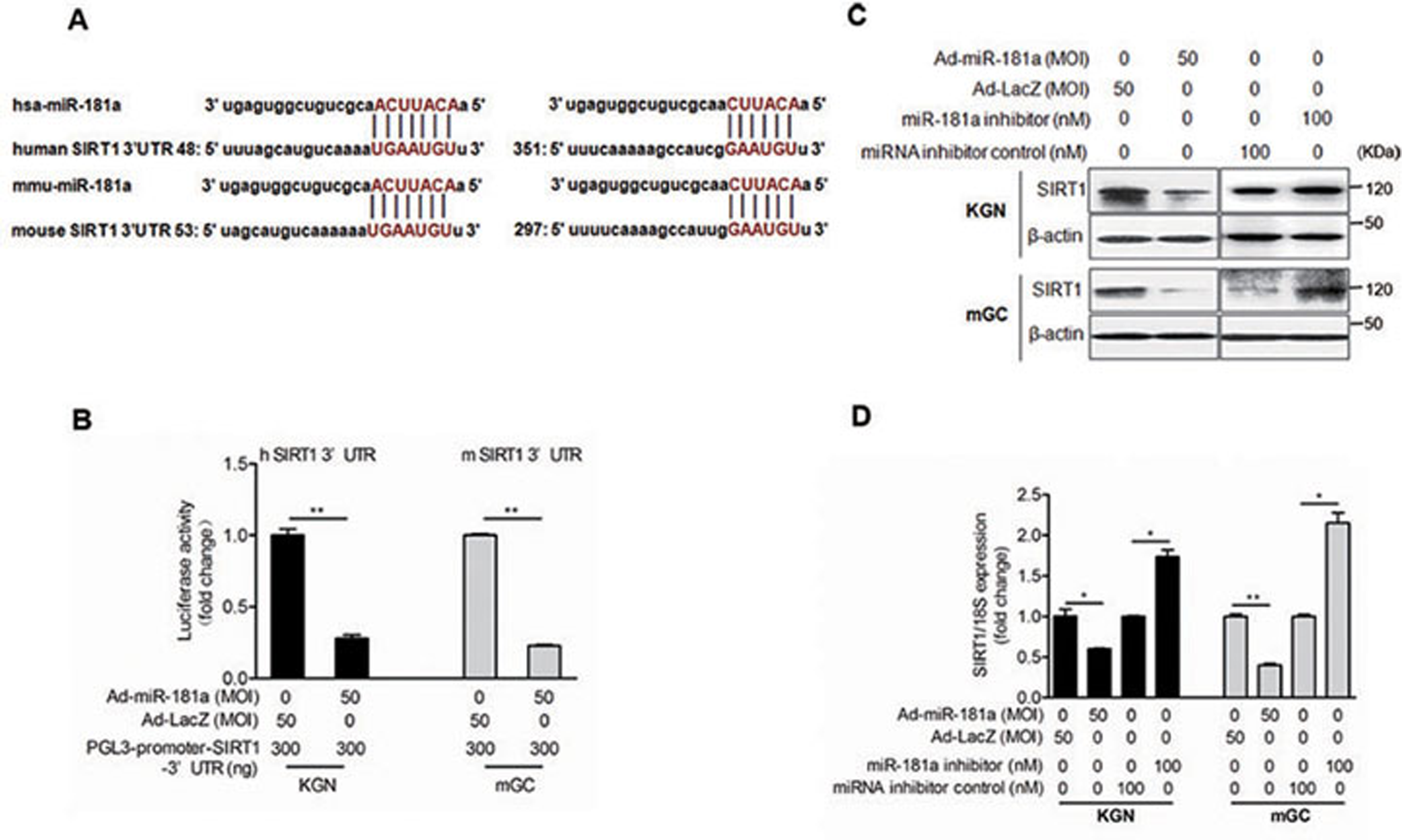

Supplement: Supplementary Figure S2 [file cddis2017467x3.tif]

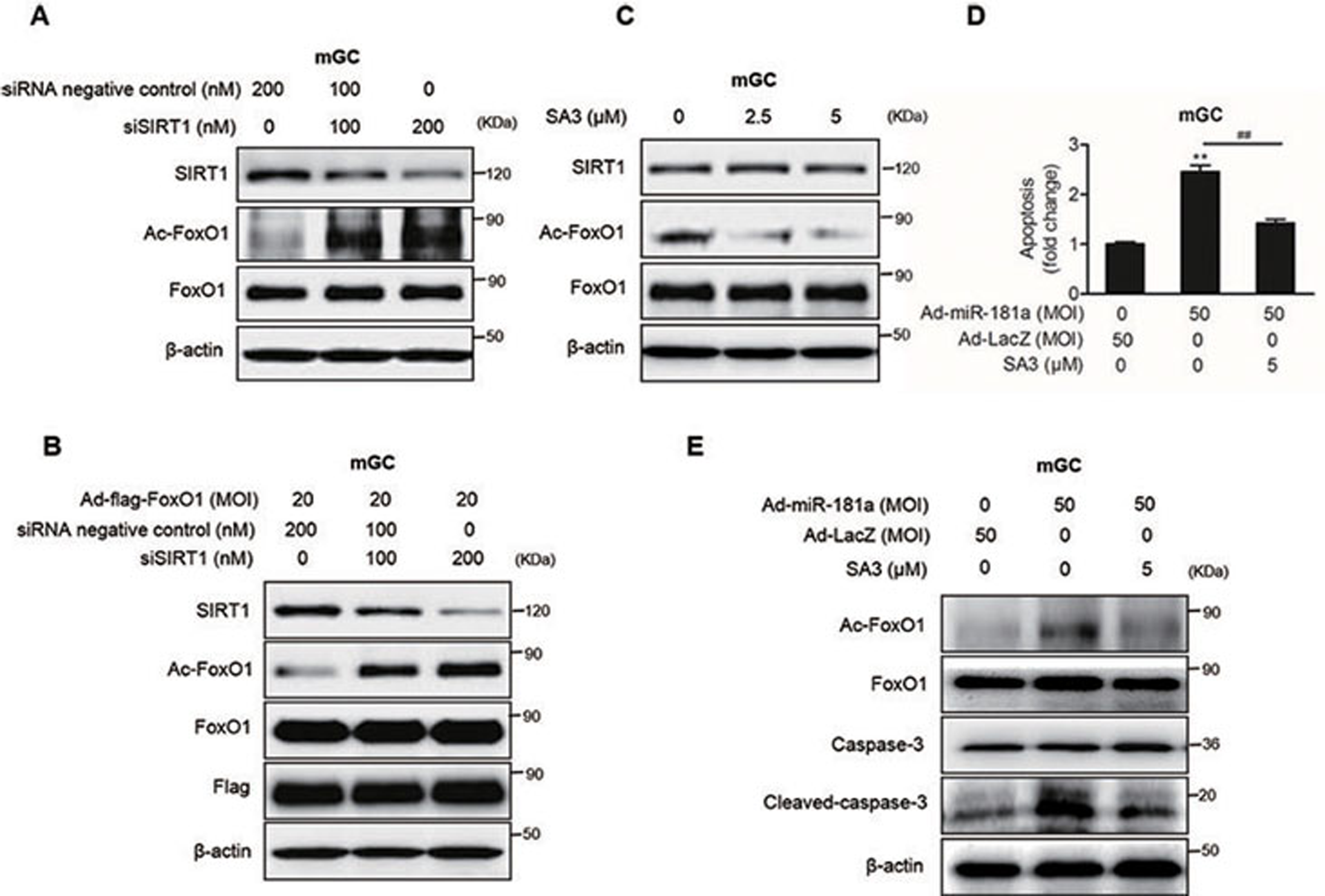

Supplement: Supplementary Figure S3 [file cddis2017467x4.tif]
